# The Interactions of Anti-HIV Pronucleotides with a Model Phospholipid Membrane

**DOI:** 10.3390/molecules29235787

**Published:** 2024-12-07

**Authors:** Monika Rojewska, Joanna Romanowska, Adam Kraszewski, Michał Sobkowski, Krystyna Prochaska

**Affiliations:** 1 Institute of Chemical Technology and Engineering, Poznan University of Technology, Berdychowo 4, 60-965 Poznań, Poland; monika.rojewska@put.poznan.pl; 2 Department of Nucleoside and Nucleotide Chemistry, Institute of Bioorganic Chemistry, Polish Academy of Science, 61-704 Poznań, Poland; msob@ibch.poznan.pl (M.S.); akad@ibch.poznan.pl (A.K.)

**Keywords:** anti-HIV pronuclotides, azidothymidine derivatives, DPPC, Langmuir monolayer, π–A isotherms, relaxation of the phospholipid film, BAM microscopy

## Abstract

Pronucleotides, after entering the cell, undergo chemical or enzymatic conversion into nucleotides with a free phosphate residue, and the released nucleoside 5′-monophosphate is then phosphorylated to the biologically active form, namely nucleoside 5′-triphosphate. The active form can inhibit HIV virus replication. For the most effective therapy, it is necessary to improve the transport of prodrugs into organelles. The introduction of new functional groups into their structure increases lipophilicity and, as a result, facilitates the interaction of pronucleotide molecules with components of biological membranes. Studies of these interactions were performed using the Langmuir technique. The prototype of the biological membrane was a thin monolayer composed of phospholipid molecules, DPPC (1,2-dipalmitoyl-*sn*-glycero-3-phosphocholine). The pronucleotides were 3′-azido-3′-deoxythymidine (AZT) analogs, formed by the phosphorylation of AZT to monophosphate (AZTMP) and containing various masking moieties that could increase their lipophilicity. Our results show the influence of the pronucleotide’s chemical structure on the fluidization of the model biomembrane. Changes in monolayer morphology in the presence of prodrugs were investigated by BAM microscopy. It was found that the incorporation of new groups into the structure of the drug as well as the concentration of AZT derivatives have a significant impact on the surface properties of the formed DPPC monolayer.

## 1. Introduction

HIV remains a major global public health issue, having claimed an estimated 42.3 million lives to date [1]. Transmission is ongoing in all countries around the world. Despite intensive research, to date, there is no effective vaccine or drug that would completely eliminate HIV from the patient’s body. Modern medicine has made it possible to maintain the virus at a level that does not cause AIDS, provided that appropriate drugs are used continuously. Therefore, the use of a multidrug approach in combating HIV is justified and necessary, and this creates a continuing need for new anti-HIV drugs.

**Figure 1:**
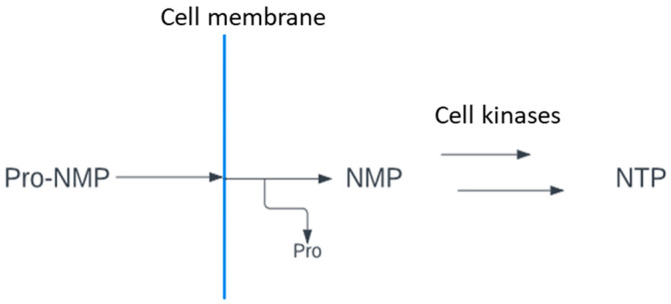
The general mode of action of pronucleotides.

Nucleoside analogs are widely applied in antiviral therapy [2,3,4]. Nucleoside/nucleotide analogs are prodrugs because after entering the cell, they must be phosphorylated by cellular kinases to the corresponding mono-, di-, and triphosphates (ddNTPs), which are proper inhibitors of viral RNA synthesis. However, some of these compounds (e.g., ddU) were found to be weak substrates for cell kinases [5], making them therapeutically useless. Due to several limitations of nucleoside-based drugs, the idea of pronucleotides arose. They were designed to bypass the first, but crucial, and at the same time, most therapeutically chimeric step of the phosphorylation of the nucleoside analog to monophosphate in the cell [6,7]. According to this concept, a prodrug would be a properly protected nucleotide-pronucleotide, from which, after entering the cell, the protective groups of the phosphate residue are removed (as a result of chemical hydrolysis or/and with the participation of cellular enzymes), and the nucleoside 5′-monophosphate is released and then phosphorylated to diphosphates and, finally, to biologically active nucleoside 5′-triphosphates [8] (Figure 1). This approach has been successfully applied for the development of antiviral drugs (e.g., for the treatment of HIV, HCV, or, recently, SARS-CoV-2) [9,10,11,12,13,14].

Unfortunately, this seemingly simple concept of pronucleotides is difficult to implement. Ideally, a prodrug should be chemically and enzymatically stable in the blood but rapidly degraded to the active parent compound once it reaches the target tissue and, most importantly, it should enter the target cell, i.e., the site of action. In the small molecule drug discovery system, there is one fundamental question that remains unanswered: how do we deliver an active drug to a molecular target? It is particularly difficult to achieve the desired distribution of the inhibitor in vivo when the target is intracellular and the mechanism of action of the inhibitor requires a chemical that has inherently poor permeability and access to the target tissue, for example, inhibitors that are predominantly charged and, therefore, highly polar at physiological pH, such as phosphates. Chemical modifications are usually intended to improve membrane permeability. For this purpose, in the concept of pronucleotides, masking groups are used on the nucleoside phosphate residue. The main task of these groups is to increase the lipophilicity of the compound so that it can penetrate the cell membranes and be metabolized inside the cell to the biologically active nucleoside 5′-triphosphate.

However, it is necessary to look closely at the biomembrane structure and possible transport mechanisms across it to efficiently design the chemical structure of the pronucleotide.

The main components of the cell membrane are lipids, proteins, and carbohydrates [15,16,17]. Lipids include phospholipids, sphingolipids, and cholesterol [8,18,19]. The first two have an amphipathic nature. They are composed of hydrophilic (“head”) and hydrophobic (“tail”) parts that determine the formation of the bilayer structure. Among the lipids, phospholipids are most common in cell membranes. They form a compact bilayer due to the presence of a phosphate group, which is connected to the hydrocarbon chains [20,21,22].

The hydrophobic tails belonging to both lipid layers come closer to each other because of van der Waals bonds, while the hydrophilic “heads” are directed toward the cytoplasm and outside the cell. This arrangement is important for the membrane—only small molecules or non-polar compounds can diffuse through the lipophilic layer [23]. The fluid mosaic model proposed by S.J. Singer and Garth L. Nicolson in 1972 allowed the visualization of many properties of the cell membrane [24]. The cell membrane consists of phospholipids that form a bilayer lipid membrane [25]. The degree of fluidization has been shown to depend on the composition and type of lipids that make up the biomembrane. The packing of hydrophobic tails plays an important role because it determines the membrane’s viscosity [20,26,27,28]. Moreover, the number of unsaturated bonds in phospholipids also has an influence on transport through the membrane. The presence of these bonds causes the hydrocarbon chain to bend and the lipids to move away from each other, which, in turn, increases the fluidity of the membrane. Saturated lipid acyl chains tend to form non-fluid tightly packed gel phases at physiological temperatures, whereas unsaturated lipid acyl chains fluidize the bilayer [29]. Lipid molecules can not only bend their non-polar tails but also rotate them around their axis [20,26,30,31,32,33]. The most widely occurring phospholipid is phosphatidylcholine, e.g., DPPC, DOPG, and DPPE [34]. Drug interactions with DPPC molecules have been shown to change the membrane permeability and determine their transport to organelles [35,36].

Biological membranes are complex structures; therefore, many simplified models of biological membranes have been developed [37,38,39]. Understanding the mechanisms of mutual interactions between membrane components, such as phospholipids and active substances, has become crucial. The Langmuir technique is one of the methods for forming and investigating biomimetic systems. The one-molecule-thick lipid film is formed as a monolayer, providing a useful model system for studying the lateral packing interactions of lipids in each leaflet of a biomembrane [40,41]. The phospholipid monolayers formed by the Langmuir technique are two-dimensional asymmetric structures with planar geometry. A Langmuir monolayer represents half of a biological membrane; therefore, it is less suited to study transmembrane processes, although it can certainly be applied to mimic processes taking place at membrane surfaces. This method allows the creation of well-defined stable structures and makes it possible to precisely control the membrane composition, the molecular areas of lipids, physical states, molecular packing, the lateral pressure of membranes, and experimental conditions, such as temperature or pH. The Langmuir technique allows the analysis of physicochemical changes that occur in the lipid membrane in the presence of therapeutic compounds at the molecular level [33,36,39,41,42,43].

One of the characterization methods of Langmuir monolayers is the analysis of the π–A isotherm. The isotherm run shows the dependence of the surface pressure (π), which is the difference between the surface tensions of the pure subphase and the subphase covered with a monolayer of phospholipids, on the average area per molecule in the thin film (A).

Introducing an amphiphilic substance under the monolayer to the subphase causes a change in the surface pressure of the lipid film and, consequently, a change in the isotherm run. The π–A isotherm provides information on the possibility of the interaction of active molecules with lipids or proteins. It determines the affinity of these substances to the components of biomembranes and the degree of incorporation into the membrane structure. The observed phase behavior of the monolayer is mainly determined by the physicochemical properties of the amphiphilic lipid, the subphase temperature, and composition. The two most commonly observed monolayer states, i.e., the liquid-expanded (LE) and liquid-condensed (LC) ones, are analogous to the liquid-crystalline and gel states in bilayers, respectively [44,45]. A lipid monolayer is also characterized by changes in terms of two-dimensional compressibility (C_s_) and is usually expressed as the surface compressional modulus (C_s_^−1^) [28,36,40]. To obtain more information about the incorporation of a given substance into a membrane, it is possible to study the relaxation of a monolayer. For this purpose, a monolayer is spread at the air–water interface and kept at a predetermined constant surface pressure. Moreover, the Langmuir technique is often supplemented with BAM microscopy, which allows us to observe changes in the membrane morphology during the compressing process.

We believe that the results obtained by the Langmuir monolayer technique will expand our understanding of the interaction mechanism of selected potential NRTIs (nucleotide reverse transcriptase inhibitors) with components of biological membranes. In our studies, the Langmuir technique was used to describe the influence of the chemical structure of prodrugs on the physicochemical properties of the phospholipid monolayer (DPPC). We believe that the knowledge gained will help us choose the right direction in the future design of new anti-HIV pronucleotides.

## 2. Results and Discussion

### 2.1. The Influence of the Chemical Structure of the Pronucleotide on the Interactions with the DPPC Monolayer

The chemical structures of the analyzed pronucleotides were modified by substituting different masking groups within the phosphate moiety, as shown in Table 1.

The application of masking groups is intended to support the transport of AZTMP through the cell membrane because the negative charge on the phosphate group prevents its easy penetration [46]. Therefore, the “pronucleotide approach” was developed, in which the phosphate group of the nucleotide analog is suitably protected and, as an uncharged form, is able to cross the cell lipid barrier. The proof of the validity of this approach is the presence of two pronucleotides (tenofovir disoproxil and tenofovir alafenamide) on the pharmaceutical market, used in the treatment of HIV and HBV. Surprisingly, later studies showed that pronucleotides do not have to be fully neutral, and those with only a partly removed negative charge showed very high antiviral activity [12,13,47,48] This implied that such compounds were able to permeate the cell membrane. To shed some light on this intriguing phenomenon, studies on the interaction of pronucleotide molecules with the DPPC monolayer as a model biomembrane were performed. Four structures were chosen as model compounds: AZT monophosphate without any protective groups (AZTMP), AZT ethyl phosphodiester (**1**), with one negative charge and a moderately lipophilic ethyl group, AZT phenyl phosphodiester (**2**), with one negative charge and a lipophilic phenyl group, and AZT phosphorodianilidate (**3**), which is uncharged and bears two lipophilic anilidate groups.

**Figure 2:**
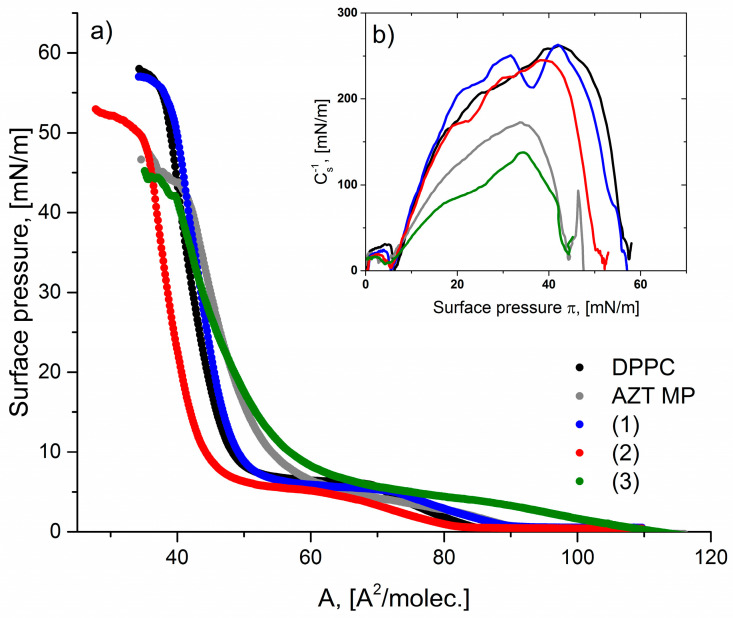
(**a**) π-A isotherms for DPPC, AZTMP and tested pronucleotides ((**1**)–(**3**)) at a concentration of 5 mg/L; (**b**) compression modulus-surface pressure (C_s_^−1^–π) graphs.

Figure 2 illustrates the π–A isotherms obtained for DPPC and pronucleotide solutions at a concentration of 5 mg/L. The π–A isotherm lift-off occurs at different area per molecule values (A_lift-off_), depending on the chemical structure of the substances tested. The values of the A_lift-off_ parameter are presented in Table 2. Azidothymidine monophosphate (AZTMP) is a compound that does not have any masking group attached to the phosphate group in its chemical structure. The addition of AZTMP to the subphase causes a slight shift of the π–A isotherm toward higher values of the average surface area per molecule in the monolayer with respect to the DPPC isotherm. The A_lift-off_ value for the DPPC/AZTMP system is ca. 92 Å^2^/molec., while for the DPPC isotherm, the A_lift-off_ oscillates around 85 Å^2^/molec. A significant effect of the π–A isotherm shift is observed for the structure with two anilidate groups (derivative (**3**), Table 1). For this system, A_lift-off_ is ca. 108 Å^2^/molec, which probably results from the planar orientation of the anilidate groups at the air–water interface. However, during the compression of the mixed monolayer DPPC/(**3**), one can observe strong condensation at high surface pressure, which was greater than 35 Å^2^/molec. (Figure 2).

A similar effect was observed by Peters et al. [49], who studied how minor differences in small molecules (isoniazid, benzhydrazide, isonicotinamide, nicotinamide, picolinamide, and benzamide) can affect their interactions with model membranes (DPPC monolayer). They have also shown that of all the compounds, only the benzamide molecule caused a decrease in area per phospholipid for DPPC at higher surface pressures (above 15–20 mN/m), suggesting that this compound either reorganized DPPC to condense further or assisted in the solvation of the DPPC molecules.

The increase in the molecular area per lipid before compressing the monolayer was due to the insertion of the benzene rings with a characteristic size and hydrophobicity; therefore, it is expected that derivative (**3**) can also significantly influence the molecular packing. The p-orbitals of the carbon atoms overlap to produce six p molecular orbitals that extend around the benzene ring. Due to their polar nature, the benzene groups interact with the polar subphase and are hydrated by water molecules. Molecular dynamics studies [50] have proven that most of the benzene rings are hydrated due to their polar nature, while the degree of hydration gradually decreases along the carbon chain. Only a small fraction of the water molecules penetrate the hydrocarbon tail part, suggesting that head groups and benzene ring groups help water molecules penetrate the monolayer film.

The DPPC isotherm has a characteristic phase transition region in the pressure range of 5–7 mN/m. The surface pressure value does not change with the compression of the DPPC monolayer, indicating the coexistence of two phases, i.e., LE and LC, in the formed lipid film [51]. In the LC/LE phase coexistence region, the DPPC film becomes a mesh of finely divided LC/LE domains. This specific structure of the film has been shown to be associated with density fluctuations, which may contribute to the flexibility and strength of the monolayer of lipids [52]. This characteristic transition is also clearly visible as a horizontal plateau in the run of the π–A isotherm obtained for the DPPC/AZTMP, DPPC/(**1**), and DPPC/(**2**) systems. No plateau can be seen in the course of the π–A isotherm for derivative (**3**). The addition of this compound to the subphase causes a stronger fluidization of the phospholipid monolayer. The presence of derivative (**3**) impacts the interactions between DPPC molecules, which is reflected in the change in the π–A isotherms run (Figure 2a). Cutro et al. [53] have suggested that this behavior can be ascribed to a different conformation of the phenyl group on the aqueous phase. They have found that phenyl group inserts in open spaces in the DPPC monolayers result in a higher area per lipid and lower compressibility. This insertion would account for the partial disappearance of the coexistence in the DPPC curves, as shown for derivative (**2**), and the total disappearance for derivative (**3**) (Figure 2a).

Dynarowicz-Łątka et al. [54] also did not observe a plateau for polyphenyl carboxylic acid (PTCA) after it was substituted by the p-nitrophenyl or bi-phenyl groups. Moreover, the presence of these groups also increased the molecular area compared to that of the parent PTCA. This effect is very similar to that observed for our systems in comparison to that observed for AZTMP molecules. We suppose that the aromatic rings can rotate with respect to each other, thus adopting different conformations. The bond connecting the phenyl groups is essentially a single bond with some double bond character [55]. The variation of a single–double bond character has been reported to depend on the presence of electron-withdrawing/electron-releasing substituents on the phenyl ring and may restrict the rotation of phenyls [53]. The phenyl group can occupy a large area at the interface at the beginning of the monolayer compression. Electrostatic interactions between the phenyl ring and water molecules causes the ring to be orientated horizontally at the interface. The rings change configuration and strongly interact with lipids during the compression of the mixed monolayer. Cutro et al. [51] have found that the interaction of phenyl with phospholipids in the interface regions generates a reconfiguration of the lipid arrangement with areas of higher lipid packing. This new arrangement in the monolayer causes the existence of a higher orientation of dipoles of lipid and water molecules, contributing to a higher overall dipole moment. They also proposed that phenyl groups may organize on the surface of the lipid arrangements, suggesting that a film is adsorbed on the lipids rather than being inserted at pH = 7.3. According to the results obtained by Saha [56], the phenyl molecules interact mostly with the head group of the lipid monolayer without directly affecting the hydrophobic interaction with the tail region at neutral pH. Saha et al. have observed a decrease in the surface pressure at neutral pH, implying an increase in the surface tension toward values of pure water. They suggested that condensation of lipids occurs to some extent on the surface, exposing water regions. Thus, it implies that the Phe (phenyl) molecules interact with the DPPC monolayer in such a manner that the DPPC molecules come closer, creating some vacant space for additional water molecules to be exposed. Based on our results, one can conclude that π–A curves obtained for mixed monolayers containing derivatives with phenyl groups ((**2**) or (**3**)) can be compressed to a lower available area at the interface in comparison to the DPPC monolayer. During compression, water molecules can be pushed into the subphase, and the highly condensed lipids will take up much less space at the available interface area.

The parameters obtained for systems with AZTMP or derivative (**1**) have shown that the incorporation of the ethyl group into pronucleotide molecules has a slight impact on the physicochemical properties of the monolayer of lipids. The collapse parameters have similar values for only the DPPC and DPPC/AZTMP systems, but for derivative (**1**), the collapse point occurred for the lower value of the surface pressure. The impact of chemical structure on the physical properties of the membrane has been observed for derivatives with one or two phenyl groups. For derivatives (**2**) and (**3**), lower values of the collapse points were reached compared to the DPPC monolayer (Table 2). This may confirm that phenyl groups strongly interact with DPPC molecules, disturbing the stability of the lipid monolayer. In Figure 2b, the compressibility modulus values and their variations as a function of the surface pressure have been presented. The compressibility modulus gives a quantitative measure of the state of the monolayer. The C_s_^−1^ value has indicated that the mixed monolayer formed by DPPC and derivative (**3**) molecules is not densely packed. We suppose that this effect is a consequence of the specific arrangement of two anilidate groups at the interface.

The hydrophobicity of a phenyl group is comparable to that of four methylene groups [52]. Therefore, we expected (**2**) and (**3**) to form more stable monolayers due to enhanced hydrophobicity, in contrast to the AZTMP molecule. Moreover, the introduction of polar groups such as nitro or amino groups into the phenyl ring allows for the disruption of the hydrophilic–hydrophobic balance that exists in the AZTMP molecule. Generally, the addition of a polar group can increase the affinity for the model cell membrane formed by DPPC.

**Figure 3:**
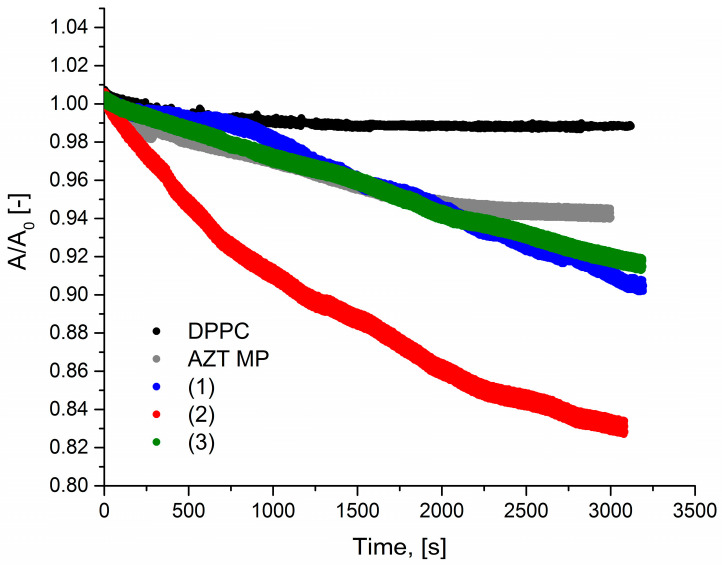
Relaxation curves for AZTMP and derivatives (**1**)–(**3**) (c = 5 mg/L) pumped underneath the DPPC monolayer.

The next studies included an analysis of the relaxation of the DPPC monolayer in the presence of pronucleotide solutions. Pumping these solutions under the DPPC monolayer caused its partial degradation. The lipid film degradation was similar for almost all derivatives considered and led to a loss of ca. 10% of the particles from the interface after 50 min. Figure 3 presents the relaxation curves for the investigated systems.

In our study, we investigated how the small difference in the chemical structure of the AZTMP derivative impacts the model membrane. Thus, we chose two derivatives with polar ((**1**) and (**2**)) and uncharged molecules (**3**). As presented in Figure 3, the run of the relaxation curve for derivative (**2**) differed significantly from the other systems. The degradation process was very intensive because 10% of the film was lost after 15 min, and finally, 20% of the film was degraded after 50 min. The molecules of derivative (**2**) interacted particularly strongly with the lipid film in contrast to derivative (**3**).

### 2.2. The Impact of the Concentration of Pronucleotides on the DPPC Monolayer

**Figure 4:**
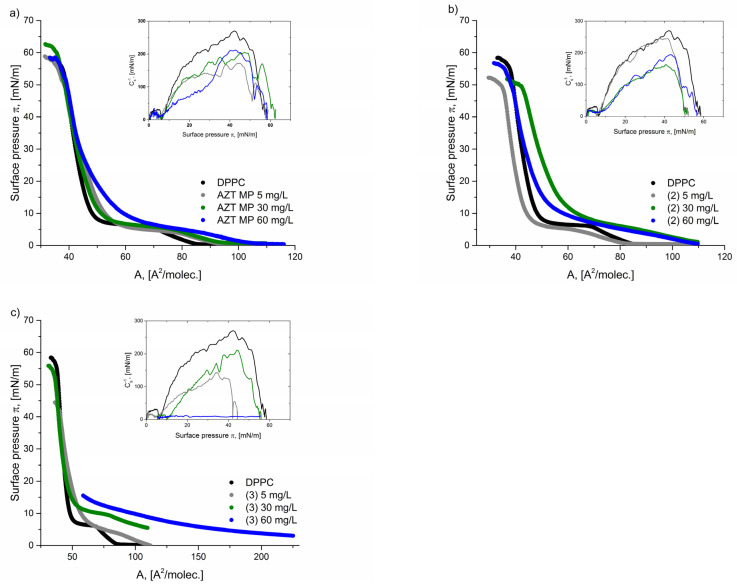
Surface pressure–area per molecule (π–A) isotherms and compression modulus–surface pressure (C_s_^−1^–π) insert graphs for the analyzed systems: (**a**) AZTMP, (**b**) derivative (**2**), and (**c**) derivative (**3**) at different concentrations: 5, 30, and 60 mg/L.

The effect of the presence of pronucleotides in the subphase on the formation of a mixed monolayer was studied for three different compounds: AZTMP, (**2**), and (**3**) at concentrations of 5 mg/L, 30 mg/L, and 60 mg/L, respectively. Figure 4 shows the π–A curves for DPPC molecules with various concentrations of the derivatives.

For all derivatives, one can observe a positive correlation, e.g., a higher concentration of the pronucleotide in the subphase results in a greater lift-off mean area occupied by the molecules at the interface (A). Increasing the concentration of derivatives in the subphase allows more molecules to be adsorbed at the air–water interface, and, simultaneously, pronucleotides interact strongly with phospholipids, thus changing the course of π–A isotherms. For derivative (**3**), its higher concentration (60 mg/L) was found to change the run of π–A isotherms dramatically. The high concentration of derivative (**3**) caused a higher initial surface pressure of the subphase. This means that derivative (**3**) molecules at 60 mg/L characterize the surface activity well, and they transfer to the interface quickly. The mixed monolayer containing DPPC and the molecules of derivative (**3**) could not be compressed to the collapse point due to experimental equipment limitations. Therefore, it only included the maximum parameters obtained for this system (Table 3).

Based on our results, it can be seen that the presence of derivative (**3**) reduces the monolayer stability, regardless of the concentration, and forms a film with the collapse at a lower surface pressure. However, we did not observe a positive correlation between the concentration of pronucleotides in the subphase and the stability of the formed film. It can only be stated that a higher concentration of pronucleotides enhances stronger interactions with DPPC molecules, which leads to the characteristic plateau disappearance of the lipid film. The plateau completely disappeared in the presence of derivative (**3**) at a concentration of 60 mg/L.

The concentration of pronucleotides affected the compressibility of the mixed films created. Generally, less packed films were obtained for lower concentrations of drugs, i.e., 5 or 30 mg/L. The most expanded monolayer was formed by the DPPC/(**3**) system at 5 mg/L (C_s_^−1^ = 142 mN/m).

**Figure 5:**
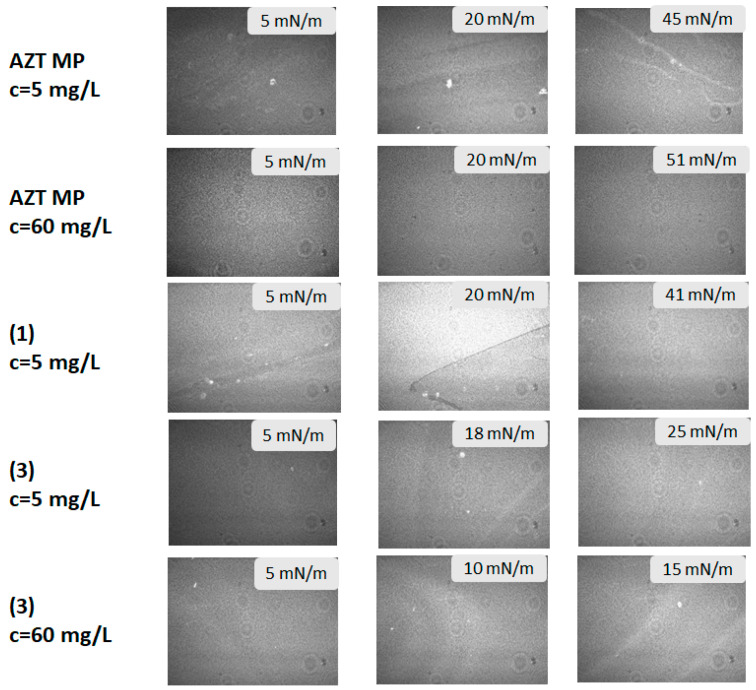
BAM images for mixed monolayers DPPC and DPPC/pronucleotides forming during compression. The size of the image is 3.6 × 4.0 mm.

The Brewster angle micrographs (BAM) of the expanded phase to the condensed phase transition in mixed systems containing pronucleotides and DPPC molecules are shown in Figure 5.

The images for the DPPC with AZTMP, DPPC/(**1**), and DPPC/(**3**) systems indicate the formation of mixed films with different morphology. The DPPC/AZTMP mixed monolayer had a morphology similar to that of the DPPC film. The formation of three-dimensional structures was visible close to the collapse point. In the case of a much higher concentration (at 60 mg/L), the aggregates were not visible. However, the coexistence of two phases, i.e., liquid and condensed, is clearly visible for the system containing derivative (**1**), especially at a surface pressure of 20 mN/m. Further compression caused this effect to disappear. The mixed monolayer consisting of DPPC and derivative (**3**) molecules formed the film with the two phases co-existing and was visible all the time during the compression of the film. The influence of concentration on film morphology was particularly visible for the mixture containing AZTMP molecules. A 12-fold increase in the particle concentration of AZTMP in the subphase caused the formation of a densely packed and uniform film at the beginning of the condensing process. As a result, the created film was so stable that the morphology did not change during compression, opposite to the monolayer formed by a lower amount of AZTMP molecules.

**Figure 6:**
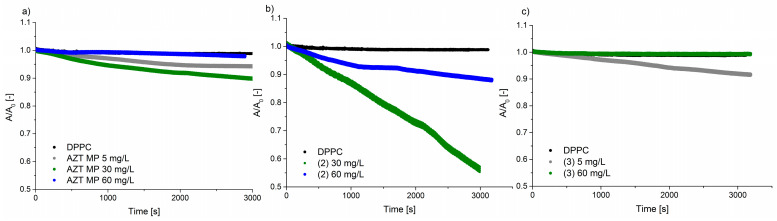
Impact of derivative concentrations on DPPC monolayer relaxation: (**a**) AZTMP, (**b**) derivative (**2**), and (**c**) derivative (**3**).

Figure 6 shows the effect of pronucleotide concentration on DPPC monolayer relaxation. It can be observed that at the middle concentration (30 mg/L), the drug interacted strongly with the lipid film and caused the desorption of the lipids into the subphase. However, the addition of pronucleotides at a concentration of 60 mg/L affected the stability more strongly than at lower concentrations.

The molecules of AZTMP are electrically charged; so, it is suspected that the polar heads of the phospholipid and the negatively charged phosphate group of AZTMP attracted each other. It is possible that as a result of electrostatic interactions, AZTMP molecules were located under the phospholipid molecules, formed the supported layer, and enhanced the stability of the DPPC monolayer. The same effect was observed for molecules of derivative (**2**). However, for a high concentration of derivative (**3**), one can see a slightly small increase in the initial value of A/A_0_ (ca. 1%), which can indicate the incorporation of pronucleotides into the DPPC monolayer.

## 3. Materials and Methods

### 3.1. Structures of Pronucleotides

For the purpose of these studies, we chose 3′-azido-3′-deoxythymidine (AZT) analogs as model compounds. AZT (zidovudine, Retrovir^®^) was the first drug approved for clinical use by the FDA (1987) for AIDS patients [57], and although it is still used today, pronucleotides containing this nucleoside analog in their structure are preferred [58]. All these compounds were obtained using previously published methods [59,60,61,62]. The structures of these prodrugs were chosen so that they differed in the number of charges and the lipophilic–hydrophilic nature of the masking groups (Table 1).

### 3.2. Chemicals

1,2-dipalmitoyl-sn-glycero-3-phosphocholine (DPPC, 99%; from Sigma Aldrich, Poznan, Poland) was used as the lipid film-forming substance. Chloroform of high-purity Uvasol (Merck, Warszawa, Poland) was applied to prepare the Langmuir monolayer.

### 3.3. Methods

#### 3.3.1. π–A Isotherms

The subphase was a water solution containing AZTMP and derivatives with appropriate concentrations. Ultrapure water produced by a PureLab Classic (ELGA, Poznan, Poland) water purification system coupled with a Milli-Q water purification system (resistivity: 18.2 MΩ∙cm) was used as a subphase. The Teflon trough and probes were washed and rinsed with isopropanol and ultrapure water. The subphase was placed in a Teflon trough (KSV Nima, Helsinki, Finland) with a surface area of 238 cm^2^. After that, the solution of DPPC with a concentration of 1 mg/L was spread on the subphase by a microsyringe (25 µL). Then, the chloroform from the interface was left to evaporate for 15 min. The monolayer was compressed by symmetrical movement of the barriers with a velocity of 10 mm/min. During the measurements, the temperature was kept constant at 25.0 ± 0.1 °C with a Julabo circulator. The surface pressure of the floating monolayer was measured to an accuracy of 0.1 mN/m using a Wilhelmy plate connected to an electrobalance. The surface pressure π (mN/m) was measured as a function of the area per DPPC molecule A (Å^2^/molec.). The compression modulus values, C_s_^−1^ = f(A), were calculated directly based on the π–A isotherm. The modulus is defined as follows [63]:

C_s_**^−^**^1^ = −A∙(^dπ^⁄_dA_)

The C_s_^−1^ values provide information on the physical state of monolayers strictly associated with the ordering and packing of molecules at the air–water interface. The value of C_s_^−1^ is assumed to be zero for the pure air–water interface and increases with the presence of active substances at the interface. A higher compression modulus value corresponds to a less compressible membrane. According to the Davies and Rideal classification [63], the gas state (G) is in the range of 0–12.5 mN/m, and the liquid-expanded (LE) state is characterized by the C_s_^−1^ modulus values between 12.5 and 50 mN/m, while the liquid-condensed (LC) state ranges between 100 and 250 mN/m. The C_s_^−1^ values above 250 mN/m refer to a solid state (S) of the monolayer. Each experiment was repeated at least three times to ensure the reproducibility of the curves to ±2 Å^2^.

#### 3.3.2. Relaxation Studies

Relaxation of the DPPC film was observed when additional derivative molecules were injected into the monolayer using a peristaltic pump (MINIPULS 3, Gilson, Middleton, WI, USA). Relaxation experiments were performed for the pure DPPC monolayer and mixed systems: DPPC/derivatives of pronucleotides. The first DPPC solution (20 µL) was spread on the air–water interface and compressed to a desired surface pressure of 30 mN/m. This surface pressure value refers to the surface pressure of the natural membrane [64,65]. After that, the pronucleotide solution was pumped underneath the DPPC film to the subphase. The flow rate set on the peristaltic pump was 13 mL/min. The pump was turned off after pumping 130 mL of pronucleotide solution, and the relaxation process was continued while maintaining a film pressure of 30 mN/m. The observed changes in the mean area per DPPC molecule were recorded over time: A = f(t). The results are presented as A/A_0_, which is the ratio of the actual area per molecule in time t to the initial area per molecule before pumping the substance (for the DPPC monolayer with π = 30 mN/m). The temperature of the experiments (25 °C) was kept constant and controlled during measurements with a Julabo F-12 circulator (Cole-Parmer, Wertheim-Mondfel, Germany).

#### 3.3.3. Brewster Angle Microscopy

Brewster angle microscopy (MicroBAM; KSV Nima, Espoo, Finland) was used to visualize the monolayer morphology. The images were captured during monolayer compression. A black glass plate was placed under the subphase to absorb the refracted beam. The camera had a field of view of 3.6 × 4.0 mm, with a resolution of approx. 6 microns per pixel.

## 4. Conclusions

The occurrence of interactions between the DPPC molecules and the AZTMP derivatives has been confirmed by the runs of π–A isotherms and the relaxation experiments for the DPPC monolayer in the presence of the considered pronucleotides. The AZTMP molecule was substituted with masking groups, and the impact of this modification on the interactions with the phospholipid monolayer was then analyzed. We assumed that the substituted AZTMP molecule with functional groups would increase the amphiphilic character of the derivative because of the partial or total elimination of the negative electrostatic surface charge. The best results were obtained when two anilidate groups were introduced into the AZTMP molecule (compound (**3**)). This derivative showed great surface activity and the strongest fluidization of the DPPC film at low concentrations. However, derivative (**3**) with DPPC molecules also formed a tightly packed film at higher concentrations. The concentration of pronucleotides was shown to strongly affect the surface properties of the phospholipid film. Masking AZTMP with anilidate groups seems to be the proper step toward the preparation of more effective pronucleotides. The trend observed in this study indicates that these molecules will probably penetrate cell membranes at higher concentrations. Thus, they are promising compounds for drug transport through model lipid membranes. However, these studies must be carried out for other components of cell membranes, such as sterols or other lipids. The presented prototype of the cell membrane is very simplified, and the experiments performed typically have a cognitive dimension, providing a direction for further, much more complex research.

## Figures and Tables

**Table 1:** Chemical structures of AZT derivatives.

Substance		Chemical Structure	Molar Mass [g/mol]
**AZTMP**	C_10_H_12_N_5_O_7_P^2−^ Na^+^	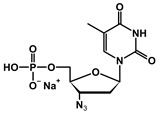	M = 345.2 M_Na+_ = 23
**(1)**	C_12_H_17_N_5_O_7_P^−^ Na^+^	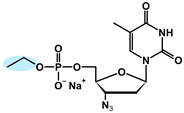	M = 374.3 M_Na+_ = 23
**(2)**	C_16_H_17_N_5_O_7_P^−^ Na^+^	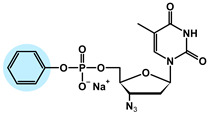	M = 422.3 M_Na+_ = 23
**(3)**	C_22_H_24_N_7_O_5_P	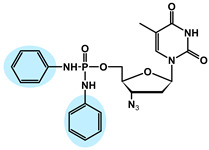	M = 497.45

**Table 2:** The characteristic parameters of π–A isotherms: A_lift-off_—lift-off area of surface pressure, A_collapse_—area corresponding to the monolayer collapse, π_collapse_—collapse pressure [mN/m], max. C_s_^−1^—maximum value of the compression modulus [mN/m], and π_max_—maximum values refers to C_s_^−1^_max_.

	A_lift-off_ [Å^2^/molec.]	A_collapse_ [Å^2^/molec.]	π_collapse_ [mN/m]	A_max_ [Å^2^/molec.]	π_max_ [mN/m]	C_s_^−1^_max_ [mN/m]
DPPC	85.1	32.9	58.4	40.2	42.3	270.5
DPPC/AZTMP	92.2	31.5	58.9	39.8	44.8	171.8
DPPC/(**1**)	88.2	32.8	56.9	41.7	40.2	264.8
DPPC/(**2**)	82.4	27.7	53.0	36.8	37.2	245.3
DPPC/(**3**)	108.1	36.3	44.5	42.3	34.6	142.1

**Table 3:** The characteristic parameters of π–A isotherms: A_lift-off_—lift-off area of surface pressure, A_collapse_—area corresponding to the monolayer collapse, π_collapse_—collapse pressure [mN/m], max. C_s_^−1^—maximum value of the compression modulus [mN/m], A_max_, and π_max_—maximum values refer to max. C_s_^−1^.

Substance	Concentration [mg/L]	A_lift-off_ [Å^2^/molec.]	π_collapse_ [mN/m]	A_collapse_ [Å^2^/molec.]	C_s_^−1^_max_ [mN/m]	A_max_ [Å^2^/molec.]	π_max_ [mN/m]
DPPC	-	85	58.4	32.9	270.5	40.2	42.3
AZTMP	5	92	58.9	31.5	171.8	39.8	44.7
	30	94	62.6	31.6	205.1	39.0	47.2
	60	102	58.5	33.2	212.2	40.8	42.7
(**2**)	5	82	53.0	27.7	245.2	36.8	37.2
	30	110	51.8	36.7	163.7	45.4	40.7
	60	109	56.8	31.7	195.0	40.2	43.2
(**3**)	5	108	44.5	36.3	142.1	42.3	34.6
	30	110	55.9	31.0	211.2	38.3	44.1
	60	222	15.5 *	58.8 *	58.8	25.7	15.5

* value corresponds to the available maximum compression.

## Data Availability

All data can be obtained from the authors upon request.
